# Design and Fabrication of Double-Layer Crossed Si Microchannel Structure

**DOI:** 10.3390/mi12121557

**Published:** 2021-12-14

**Authors:** Yipeng Wang, Weijian Zhou, Tieying Ma

**Affiliations:** College of Optical and Electronic Technology, China Jiliang University, Hangzhou 310013, China; ypwang2021@sinano.ac.cn (Y.W.); Zhouweijian18@163.com (W.Z.)

**Keywords:** MEMS, silicon, wet etching, microchannel

## Abstract

A four-step etching method is used to prepare the double-layer cross Si microchannel structure. In the first etching step, a <100> V-groove structure is etched on (100) silicon, and the top channel is formed after thermal oxidation with the depth of the channel and the slope of its sidewall being modulated by the etching time. The second etching step is to form a sinking substrate, and then the third step is to etch the bottom channel at 90° (<100> direction) and 45° (<110> direction) with the top channel, respectively. Hence, the bottom channel on the sink substrate is half-buried into the top channel. Undercut characteristic of 25% TMAH is used to perform the fourth step, etching through the overlapping part of the two layers of channels to form a double-layer microchannel structure. Different from the traditional single-layer microchannels, the double-layer crossed microchannels are prepared by the four-step etching method intersect in space but are not connected, which has structural advantages. Finally, when the angle between the top and bottom is 90°, the root cutting time at the intersection is up to 6 h, making the width of the bottom channel 4–5 times that of the top channel. When the angle between the top and bottom is 45°, the root cutting time at the intersection is only 4 h, and due to the corrosion along (111), the corrosion speed of the sidewall is very slow and the consistency of the width of the upper and lower channels is better than 90° after the end. Compared with the same-plane cross channel structure, the semiburied microchannel structure avoids the V-shaped path at the intersection, and the fluid can pass through the bottom channel in a straight line and cross with the top channel without overlapping, which has a structural advantage. If applied to microfluidic technology, high-efficiency delivery of two substances can be carried out independently in the same area; if applied to microchannel heat dissipation technology, the heat conduction area of the fluid can be doubled under the same heat dissipation area, thereby increasing the heat dissipation efficiency.

## 1. Introduction

Microchannels can be widely used in microchannel plates (MCP), microchemical equipment (micro heat exchangers, microreactors, micromixers, micro separators, etc.), total microanalysis systems (microfluid control chips and microarray chips), micro heat conduction equipment, etc. [1,2,3]. Silicon has high mechanical strength, high purity, corrosion resistance and good heat dissipation. Most importantly, using silicon as a microchannel can be compatible with integrated circuit technology. They have been extensively developed in various fields, including microelectronic devices, microelectrodes, electrocatalytic materials, functional materials, and new energy materials [4].

**Figure 1:**
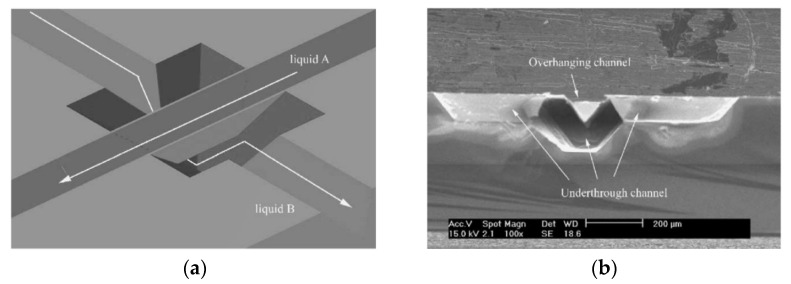
(**a**) Schematic diagram of plane crossing channel structure. (**b**) Sectional details.

Most of the Si microchannel structures reported at present are in the same layer, in the shape of “T”, “Y”, comb, cross, Serpentine, and spiral [5,6,7,8,9,10,11,12,13,14,15,16,17,18]. As the feature size of microdevices decreases and the integration degree increases, more complex design and more extended wiring are required in single-layer microchannels, which will affect the efficiency of the device operation. Jae Wan Kwon and Eun Sok Kim [19] proposed a cross-channel structure, in which a deep cavity was formed at the intersection by the undercut method so that two crossing channels in the same plane could be independently routed (Figure 1a). Compared with the single-layer channel, the design of a double-layer cross channel enables transmission in different directions in the same space, shortens the path and improves efficiency. It lays the experimental foundation for the preparation of high-efficiency microfluidics and micro radiator. However, it is slightly inadequate: in order not to connect with each other, the bottom channel must go from one end of the top channel to the other through a V-shaped path (Figure 1b), rather than straight through; the channel opening at the bottom intersection must be larger than the opening at the top to undercut. This will significantly affect the uniformity of the flow velocity of the underlying channel.

In this paper, a semiburied double-layer cross microchannel structure based on a sunken substrate is proposed. The substrate is sunk after the preparation of the top channel, the bottom channel is partially buried in the top channel, and the intersection of the bottom and top channels is directly tunneled by the undercut method. Compared with the double-layer channel reported above, the transmission path of the bottom channel in this paper is a straight line in the same horizontal plane, and the fluid flow velocity is more uniform throughout the channel. In the future, new technologies can be explored on channel shape and channel materials to prepare a more stable and superior upper and lower cross-channel structure.

## 2. Design

**Figure 2:**
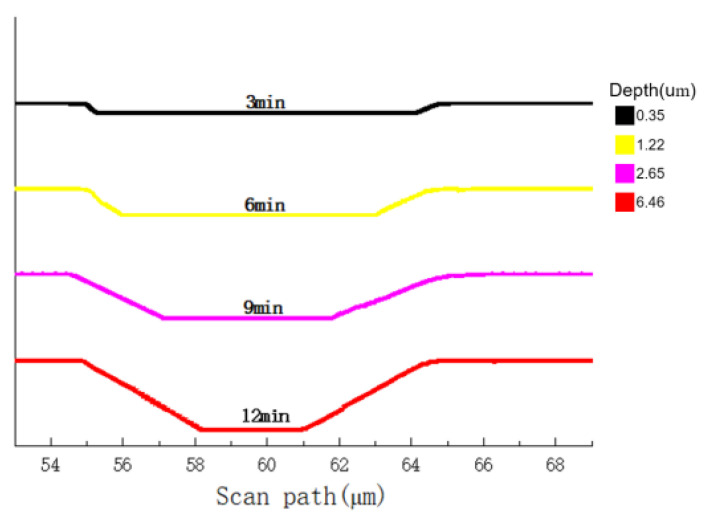
Scanning diagrams of channel cross-section steps with different etching times.

The double-layer microchannel structure is fabricated on (100) single crystal silicon wafers. The <100> top channel with a V-shaped cross-section is prepared by anisotropic wet etching. The etching solution KOH + IPA is along the <100> direction on both sides for side etching. The depth of the groove and the slope of V-shaped sidewalls can be modulated by etching time. Four sets of samples are prepared with 3 min, 6 min, 9 min, 12 min etching time, respectively. According to test data of profiler, the groove depths are 0.35 μm, 1.22 μm, 2.65 μm, 6.46 μm while inclination angles of sidewalls are 82°, 70°, 54°, and 30° respectively (Figure 2). Considering cross-sectional shape on the top channel and quick release on the bottom channel, the etching time of the top channel is selected to be 12 min.

**Figure 3:**
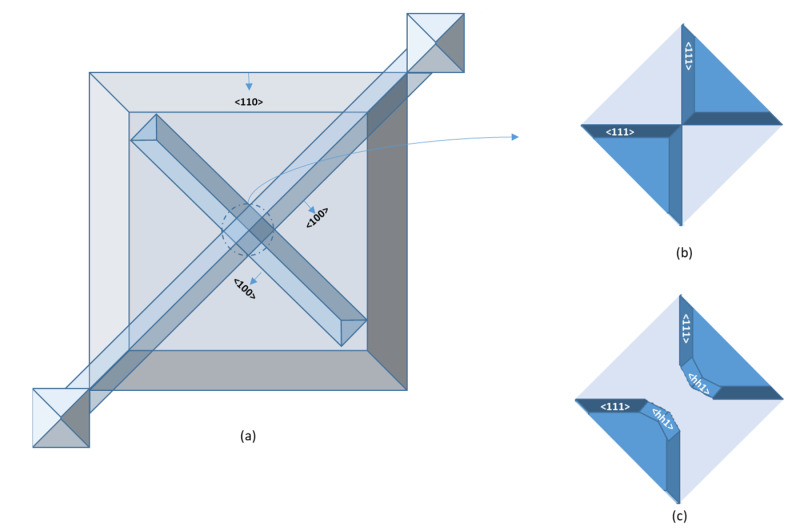
(**a**) Top view of 90° dual-channel structure. (**b**) Four self-stopping etch surfaces (111) to form two vertical angles. (**c**) Undercutting to expose the (hh1) surface.

After thermal oxidation, equilateral right triangle openings are exposed on both sides of the top groove as a cavity (Figure 3a), and the hypotenuse and right-angle sides are located in <100> and <110> directions, respectively. After the second anisotropic wet etching step, two triangle areas sinking, sinking depth can be modulated by etching time. If the etching time is long enough, etchant undercuts along <100> direction, and the top channel will be supported entirely or even suspended above the cavity, which is conducive to cut-through of the following bottom channel. However, the top channel film has no bulk Si support, easily cracked or even collapsed. Therefore, a short etching time is modulated not altogether to remove the bulk silicon under the sidewall of the top channel. Hence, the bulk silicon is used to reinforce the top channel so that the top channel is half-mounted on the cavity. Simultaneously, the etchants cut along <110> right angle side, forming four (111) faces as cavity sides. After oxidation, the bottom channel strip opening is designed at the bottom of the cavity and half-buried into the top channel. After KOH + IPA etches the bottom trench, the overlapping parts of the layers are penetrated by the good undercutting characteristics of 25% TMAH to form a double-layer microchannel structure with upper and lower crossing. In order to compare the undercutting characteristics of tunnels with different crystal directions, <100> and <110> underlying channel openings are designed, respectively. The <100> bottom channel intersects vertically with the top <100> channel. At the intersection, four self-stopping etch surfaces (111) will be fabricated to form two vertical angles (Figure 3b), and undercutting will occur to expose the (hh1) surface (Figure 3c) [20].

**Figure 4:**
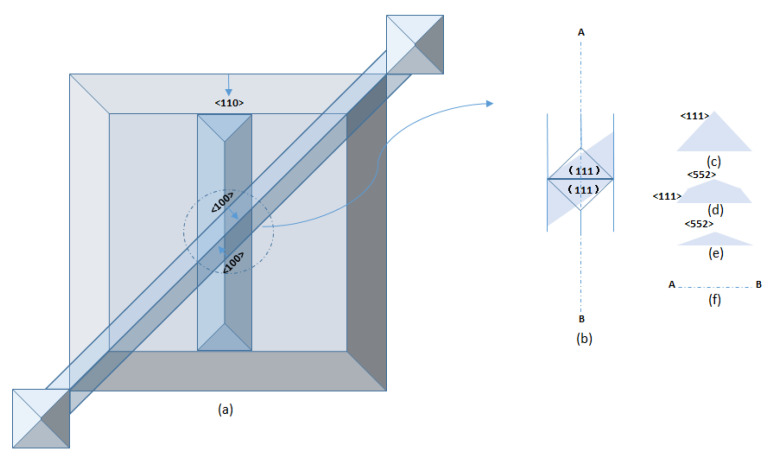
(**a**) Top view of 45° dual-channel structure (**b**) Top view of details of overlapping area. (**c**) A convex angle consisting of two (111) faces (**d**) <552> oriented planes are exposed at the convex corner formed by two (111) faces (**e**) the convex corner formed by two (552) faces (**f**) adjoining (111) planes is formed and channel is through.

The <110> bottom strip opening is at a 45° angle to the top <100> direction channel (Figure 4a). The etchants will first advance rapidly along <100> on both sides of the channel, forming a convex angle consisting of two (111) faces (Figure 4b), and then undercutting at the interface edge. Both the {552} planes and the {111} planes will be exposed, and further etching will self-stop by the formation of adjoining {111} planes (Figure 4c–f) [21].

## 3. Experimental Procedure

**Figure 5:**
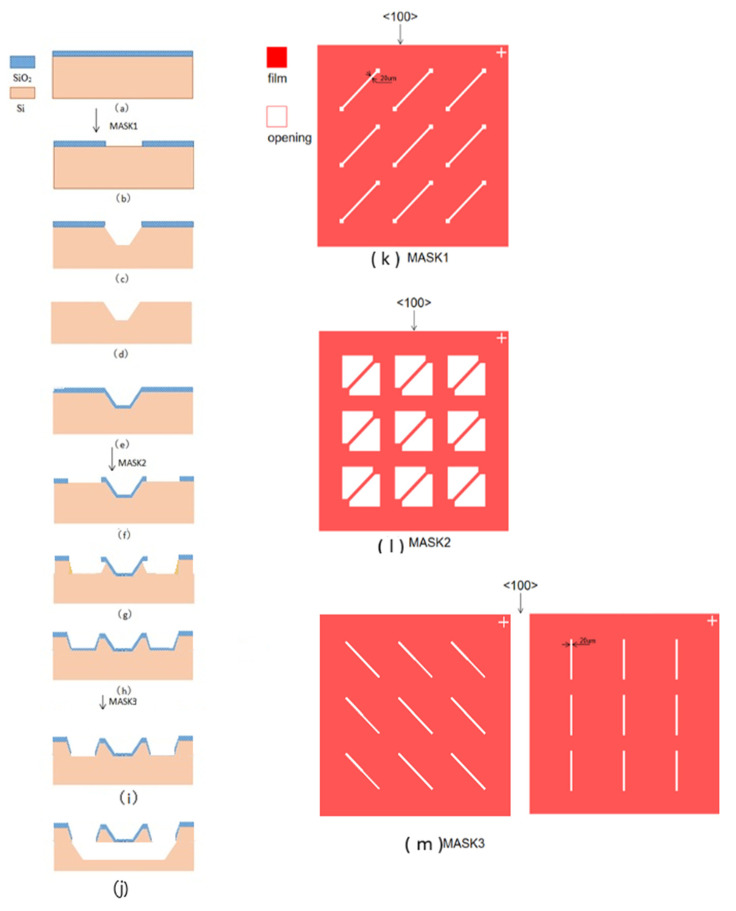
(**a**) SiO_2_ is thermally oxidized on (100) bulk silicon. (**b**) SiO_2_ is etched by BOE to form a 20 μm wide strip opening. (**c**) <100> direction V groove is etched. (**d**) Remaining SiO_2_ mask is removed. (**e**) SiO_2_ is grown again. (**f**) Two right-angle equilateral triangle openings are exposed. (**g**) Two triangle sunk areas are formed. (**h**) Regrow SiO_2_ as support layer of the top channel and the mask layer of the bottom channel. (**i**) The SiO_2_ mask is removed partly to form 20 μm wide strip openings, which are at 90° and 45° respectively with the first channel. (**j**) Bottom groove is obtained by KOH + IPA and then be run through by TMAH. (**k**) Mask No. 1 with top channel openings. (**l**) Mask No. 2 with openings of triangle sunk areas. (**m**) Mask No. 3 with bottom channel openings.

Firstly, a layer of SiO_2_ with a thickness of 2 microns is thermally oxidized on (100) bulk silicon as the mask layer (Figure 5a). After photolithography with mask No. 1 (Figure 5k), SiO_2_ is etched by BOE to form a strip opening (Figure 5b). At 80 °C, 50% KOH + IPA solution is used to get a <100> direction V groove after etching for 12 min (Figure 5c). After removing the remaining SiO_2_ mask (Figure 5d), a layer of dense SiO_2_ with a thickness of 2 microns is grown by thermal oxidation again (Figure 5e). Two right-angle equilateral triangle openings are exposed with mask No. 2 (Figure 5l) photolithography and BOE etching (Figure 5f). KOH + IPA is used for etching for 3 min to make two triangle areas sink (Figure 5g). A layer of dense SiO_2_ with a thickness of 2 microns is grown by thermal oxidation again as the support layer of the top channel and the mask layer of the bottom channel (Figure 5h). The SiO_2_ mask is removed partly with mask No. 3 (Figure 5m) with two design patterns to form strip openings (Figure 5i), which are at 90° and 45°, respectively with the first channel. Then KOH + IPA is used for etching for 12 min, and a V-shaped bottom groove is obtained. At this time, the intersection of the two layers of channels is not penetrated, and 25% TMAH is used to make the intersection run through (Figure 5j).

## 4. Results and Discussion

**Figure 6:**
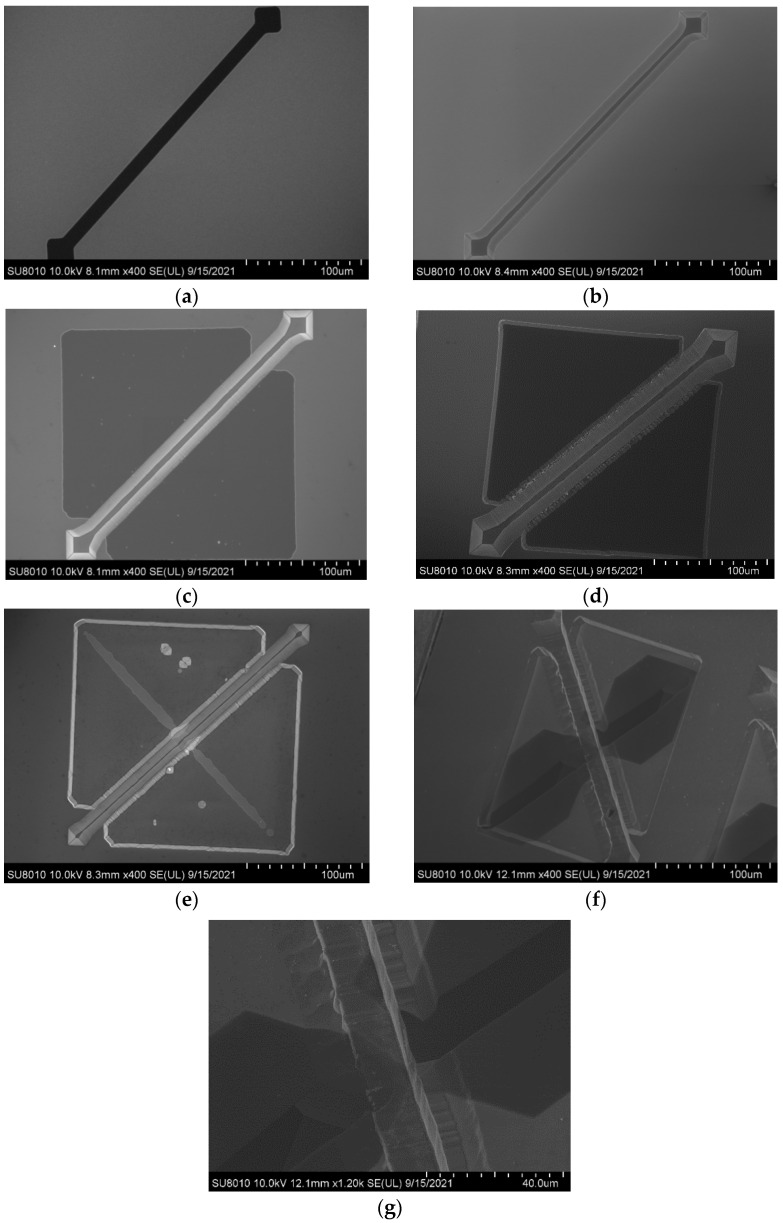
(**a**) SiO_2_ strip opening for top channel. (**b**) <100> direction V groove for top channel. (**c**) Two right-angle equilateral triangle openings. (**d**) Two triangle sunk areas. (**e**) Regrow SiO_2_ as support layer of the top channel and the mask layer of the bottom channel. (e) The 90° bottom channel strip openings with top channel. (**f**) Top view of 90° dual-channel structure. (**g**) Details of intersection at 90° dual-channel.

Since the SiO_2_ film is semitransparent, the suspended area and the adhesion area are different. The etching process can be observed in situ through a metallurgical microscope, and etching parameters can be optimized in time. Scanning electron microscopy (SEM) can be used to characterize the microstructure of the critical steps, as shown below (Figure 6a–f). When the angle between the two channels is 90°, the corresponding SEM figure is as follows. The TMAH etching solution first advances along the <100> direction at the underlying channel. After the (111) self-stop etching surfaces under both ends of the top channel meet, two opposite corners are formed and an undercut occurs, exposing the (hh1) surface. At this time, the whole bottom tunnel is connected. The long undercut time of 6 h results in a significant broadening of the bottom channel, which is even 4–5 times the width of the top channel. After release, the SiO_2_ sidewall film at the intersection of the top channel is suspended without collapse (Figure 6g).

**Figure 7:**
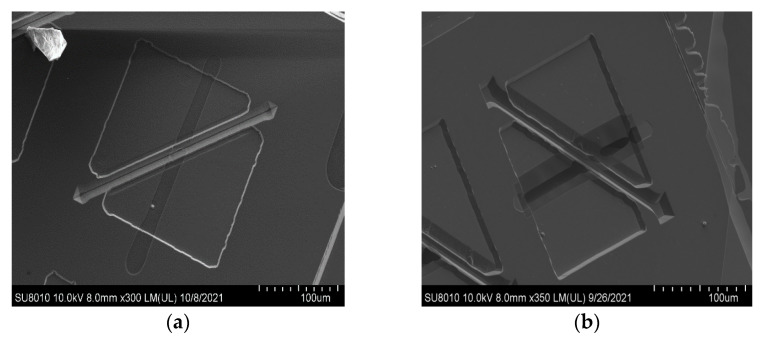
(**a**) The 45° bottom channel strip openings with top channel. (**b**) Top view of 45° dual-channel structure.

**Figure 8:**
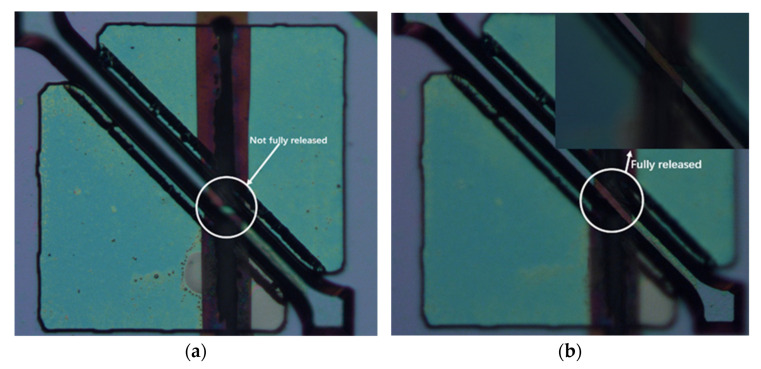
(**a**) The incomplete release of the bottom channel. (**b**) The complete release of the bottom channel, and the upper right corner of the diagram showing enlargement at the intersection of the upper and lower channels.

**Figure 9:**
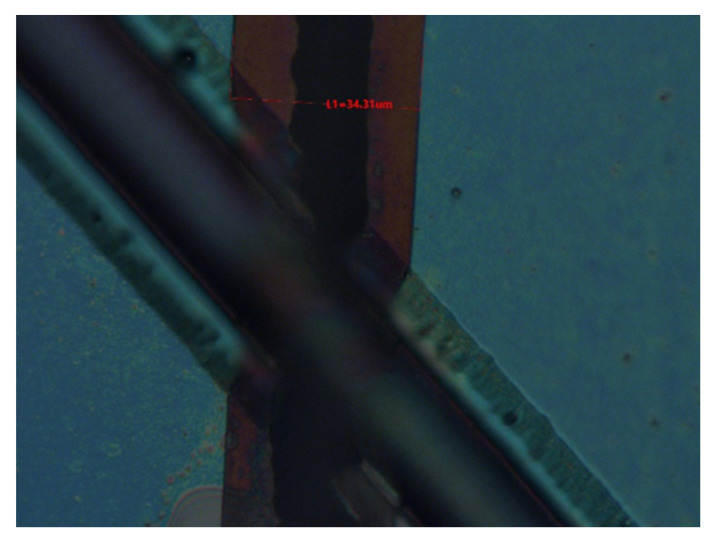
The width of bottom channel measured by metallographic microscope.

**Figure 10:**
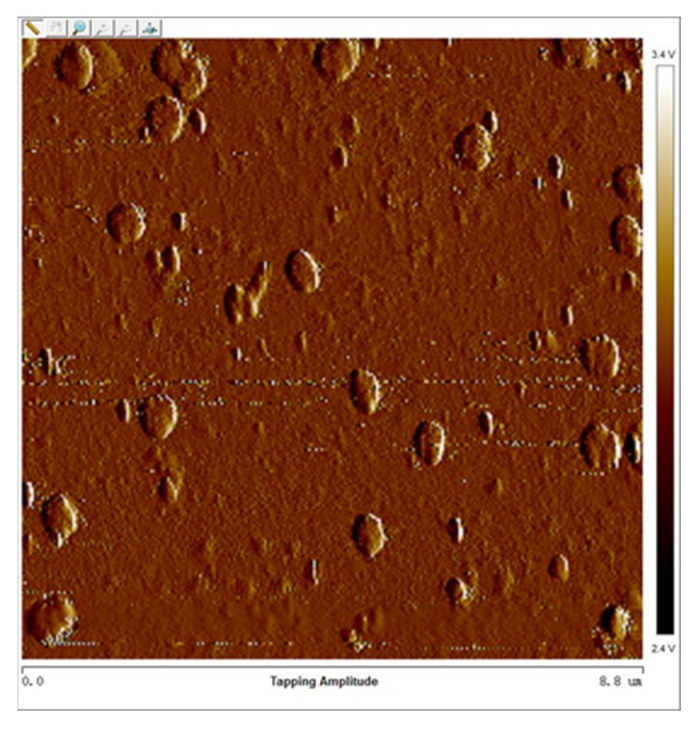
Channel roughness map by Atomic Force Imaging.

In a 90° dual-channel structure, the middle part of the bottom channel is blocked partly by the (111) slow-etched bulk Si and becomes narrow. However, due to the rapid advancement of the etchant along the <100> direction and the long undercutting time, the two ends show apparent broadening and deepening. The following opening of the bottom is designed as <110> direction, and the included angle between the two channels is 45° (Figure 7). The undercut section at the intersection transitions from (111) to (552) gradually, and finally, it is completely connected (Figure 8). However, the undercutting time is shorter than that of the 90° dual-channel, only 4 h. In addition, the (111) lateral wall of the bottom channel has a plodding advance speed after it self-stops etching, resulting in better consistency of the channel width than that of the 90°’s. In two groups of structures, the convex edge at the intersection of top channels is slightly damaged due to high steps in 3D structures, leading to the nonuniform distribution of photoresists. Hence, the photoresist covered by the convex edge is too thin, which is easy to remove and cannot fully protect the top of the first layer channel. However, the V-shaped main body of the top channel is intact, and it does not hinder the smooth passage of fluid. The width of the bottom channel measured by a metallographic microscope is about 34.31 μm (Figure 9). Since the bottom channel corrodes along <111>, the angle between the sidewall of the bottom channel and the horizontal channel is 54.7°. The calculated channel depth is 24.23 μm, the cross-section is an isosceles triangle, and the cross-section area is 415.67 μm^2^. The channel surface roughness was measured by atomic force microscopy (Figure 10), the arithmetic mean roughness (Ra) of the outline is 6.75 nm, and the root mean square roughness (Rq) of the outline is 11.5 nm.

## 5. Conclusions

Double-layer crossed Si microchannel structures are proposed and fabricated in this paper. The top channel is along the <100> direction, with its depth and inclination of the sidewall being modulated by etching time. The bottom channel is prepared on a sinking substrate and is partially buried under the top channel. For bottom channels, two directions are designed, namely the <100> direction perpendicular to the top channel and the <110> direction at a 45° angle to the top channel. The undercutting time of the <100> bottom channel is so long that the sidewalls at nonintersection are widened. However, the undercutting time of the <110> bottom channel is shorter than that of the <100> bottom channel. Moreover, due to its sidewall’s etching-stop, the width of the whole bottom channel is narrower and more uniform than that of the <100> bottom channel. Compared with the same-plane cross channel structure, the semiburied microchannel structure avoids the V-shaped path at the intersection, and the fluid can pass through the bottom channel in a straight line and cross with the top channel without overlapping, which has a structural advantage. Applied to microfluidic technology, two substances can be independently delivered in the same area and analyzed at the same time with high efficiency. Applied to microchannel heat dissipation technology, the heat conduction area of the fluid can be doubled under the same heat dissipation area, thereby increasing the heat dissipation efficiency.

## References

[1] Roos M.M., Winkler A., Nilsen M., Menzel S.B., Strehle S. (2021). Towards Green 3D-Microfabrication of Bio-MEMS Devices Using ADEX Dry Film Photoresists. Int. J. Precis. Eng. Manuf.-Green Technol..

[2] Kamat A.M., Pei Y., Jayawardhana B., Kottapalli A.G.P. (2021). Biomimetic Soft Polymer Microstructures and Piezoresistive Graphene MEMS Sensors Using Sacrificial Metal 3D Printing. ACS Appl. Mater. Interfaces.

[3] Velden G., Fan D., Staufer U. (2020). Fabrication of a microfluidic device by using two-photon lithography on a positive photoresist. Micro Nano Eng..

[4] Kaltsas G., Pagonis D.N., Nassiopoulou A.G. (2004). Planar CMOS compatible process for the fabrication of buried microchannels in Si using porous Si technology. J. Microelectromech. Syst..

[5] Ahmed H., Ramesan S., Lee L., Rezk A.R., Yeo L.Y. (2020). On-Chip Generation of Vortical Flows for Microfluidic Centrifugation. Small.

[6] Jasińska L., Malecha K. (2021). Microfluidic Modules Integrated with Microwave Components—Overview of Applications from the Perspective of Different Manufacturing Technologies. Sensors.

[7] Xu X., Huang X., Sun J., Wang R., Yao J., Han W., Wei M., Chen J., Guo J., Sun L. (2021). Recent progress of inertial microfluidic-based cell separation. Analyst.

[8] Fatehifar M., Revell A., Jabbari M. (2021). Non-Newtonian Droplet Generation in a Cross-Junction Microfluidic Channel. Polymers.

[9] Chung Y.C., Chung H.H., Lin S.H. (2021). Improvement of Temperature and Optical Power of an LED by Using Microfluidic Circulating System of Graphene Solution. Nanomaterials.

[10] Yuan C., Zhang H., Li X., Oishi M., Oshima M., Yao Q., Li F. (2021). Numerical Investigation of T-Shaped Microfluidic Oscillator with Viscoelastic Fluid. Micromachines.

[11] Fallahi H., Zhang J., Phan H.P., Nguyen N.T. (2019). Flexible Microfluidics: Fundamentals, Recent Developments, and Applications. Micromachines.

[12] Cai G., Xue L., Zhang H., Lin J. (2017). A Review on Micromixers. Micromachines.

[13] Liu C., Hu G. (2017). High-Throughput Particle Manipulation Based on Hydrodynamic Effects in Microchannels. Micromachines.

[14] Pattanayak P., Singh S.K., Gulati M., Vishwas S., Kapoor B., Chellappan D.K., Anand K., Gupta G., Jha N.K., Gupta P.K. (2021). Microfluidic chips: Recent advances, critical strategies in design, applications and future perspectives. Microfluid. Nanofluidics.

[15] Zhang J., Yan S., Yuan D., Alici G., Nguyen N.T., Warkiani M.E., Li W. (2015). Fundamentals and Applications of Inertial Microfluidics: A Review. Lab Chip.

[16] Cubaud T., Mason T.G. (2012). Interacting viscous instabilities in microfluidic systems. Soft Matter.

[17] Wang Y., Nunna B.B., Talukder N., Etienne E.E., Lee E.S. (2021). Blood Plasma Self-Separation Technologies during the Self-Driven Flow in Microfluidic Platforms. Bioengineering.

[18] Herrmann N., Neubauer P., Birkholz M. (2019). Spiral microfluidic devices for cell separation and sorting in bioprocesses. Biomicrofluidics.

[19] Kim K. (2002). Multi-level microfluidic channel routing with protected convex corners. Sens. Actuators A.

[20] Pal P., Sato K. (2015). A comprehensive review on convex and concave corners in silicon bulk micromachining based on anisotropic wet chemical etching. Micro Nano Syst. Lett..

[21] Kim H.S., Kim J.M., Bang Y.S., Song E.S., Ji C.H., Kim Y.K. (2012). Fabrication of a vertical sidewall using double-sided anisotropic etching of 100 oriented silicon. J. Micromech. Microeng..

